# Complex regulation of microRNAs in roots of competitively-grown isogenic *Nicotiana attenuata* plants with different capacities to interact with arbuscular mycorrhizal fungi

**DOI:** 10.1186/s12864-018-5338-x

**Published:** 2018-12-17

**Authors:** Priyanka Pandey, Ming Wang, Ian T. Baldwin, Shree P. Pandey, Karin Groten

**Affiliations:** 1 National Institute of Biomedical Genomics, Kalyani, West Bengal India; 2 Department of Molecular Ecology, Max Planck Institute for Chemical Ecology, Hans-Knöll-Str. 8, 07745 Jena, Germany; 3 Department of Biological Sciences, IISER Kolkata, Mohanpur, Nadia, West Bengal 741246 India

**Keywords:** microRNA, Arbuscular mycorrhiza, *Nicotiana attenuata*, Signaling, Solanaceaous species, Phosphate starvation

## Abstract

**Background:**

*Nicotiana attenuata* is an ecological model plant whose 2.57 Gb genome has recently been sequenced and assembled and for which miRNAs and their genomic locations have been identified. To understand how this plant’s miRNAs are reconfigured during plant-arbuscular mycorrhizal fungal (AMF) interactions and whether hostplant calcium- and calmodulin dependent protein kinase *(CCaMK)* expression which regulates the AMF interaction also modulates miRNAs levels and regulation, we performed a large-scale miRNA analysis of this plant-AMF interaction.

**Results:**

Next generation sequencing of miRNAs in roots of empty vector (EV) *N. attenuata* plants and an isogenic line silenced in *CCaMK* expression (ir*CCaMK*) impaired in AMF-interactions grown under competitive conditions with and without AMF inoculum revealed a total of 149 unique miRNAs: 67 conserved and 82 novel ones. The majority of the miRNAs had a length of 21 nucleotides. MiRNA abundances were highly variable ranging from 400 to more than 25,000 reads per million. The miRNA profile of ir*CCaMK* plants impaired in AMF colonization was distinct from fully AMF-functional EV plants grown in the same pot. Six conserved miRNAs were present in all conditions and accumulated differentially depending on treatment and genotype; five (miR6153, miR403a-3p, miR7122a, miR167-5p and miR482d, but not miR399a-3p) showed the highest accumulation in AMF inoculated EV plants compared to inoculated ir*CCaMK* plants. Furthermore, the accumulation patterns of sequence variants of selected conserved miRNAs showed a very distinct pattern related to AMF colonization - one variant of miR473-5p specifically accumulated in AMF-inoculated plants. Also abundances of miR403a-3p, miR171a-3p and one of the sequence variants of miR172a-3p increased in AMF-inoculated EV compared to inoculated ir*CCaM*K plants and to non-inoculated EV plants, while miR399a-3p was most strongly enriched in AMF inoculated ir*CCaMK* plants grown in competition with EV. The analysis of putative targets of selected miRNAs revealed an involvement in P starvation (miR399), phytohormone signaling (Nat-R-PN59, miR172, miR393) and defense (e.g. miR482, miR8667, Nat-R-PN-47).

**Conclusions:**

Our study demonstrates (1) a large-scale reprograming of miRNAs induced by AMF colonization and (2) that the impaired AMF signaling due to *CCaMK* silencing and the resulting reduced competitive ability of ir*CCaMK* plants play a role in modulating signal-dependent miRNA accumulation.

**Electronic supplementary material:**

The online version of this article (10.1186/s12864-018-5338-x) contains supplementary material, which is available to authorized users.

## Background

Root colonization by arbuscular mycorrhizal fungi (AMF), which belong to the phylum Glomeromycota [1], is wide-spread across almost all plant families, and it is estimated to occur in more than 70% of all terrestrial plants [2]. This interaction improves the plant’s uptake of nutrients from the soil, in particular of inorganic phosphorus and nitrogen, while in exchange, the plants provide carbohydrates to the fungus [3]. Only more recently it was also demonstrated that lipids are transferred from the plants to the fungus [4, 5]. The major site of exchange is the arbuscule, a tree-like structure within the cortical cells, which greatly enhances the interface between plant and fungus [6]. The arbuscule is surrounded by the periarbuscular membrane which contains many transporters [7]. AMF colonization can also improve the plant’s resistance against pests and pathogens and reduce drought stress [8, 9]. The communication and signaling processes between plants and AMF have been investigated in detail, and many genes of the signaling cascade required for a successful colonization of the plant root by AMF have been elucidated [10]. Fungal lipochitooligosaccharides [11] and short chain chitin oligomers [12] are recognized by the roots, presumably via receptor-like kinases, and this recognition results in phosphorylation-induced Ca spiking mediated by the cation channels Castor and Pollux and nup133 [13, 14]. The Ca^2+^ signal is decoded by a calcium and calmodulin protein dependent kinase in interaction with IPD3/Cyclops [15–17]. Additionally, DELLA proteins are part of this complex [18], and further downstream signals include a number of GRAS transcription factors such as NSP2 and RAM1, regulating among others genes that are essential for the development and functioning of arbuscules, such as STR, STR2, RAM2, vapyrin and PT4 [7, 19]. More recently, small non-coding RNAs (smRNAs) have emerged as important regulators that control plant development [20] and abiotic and biotic stress responses [21, 22]. SmRNAs are divided into two main classes, microRNAs (miRNAs) and small interfering RNAs (siRNAs) [23]. In plants, miRNAs are mainly 20 to 24 nucleotides in length with a typical stem loop structure. Precursor miRNAs are cleaved by Dicer-like enzymes (DCLs) into 5p/3p-miRNA/miRNA*-duplexes with 2 nt overhangs. After loading one strand in an effector RISC (RNA-induced silencing complex) containing Argonaute proteins (AGOs), the miRNAs mainly negatively regulate gene expression by cleavage of mRNAs or inhibition of translation [24]. MiRNAs are often found in multigene families. Many miRNAs are conserved across angiosperms and seem to play similar roles in different plant species.

MiRNAs have been reported to play a role in plant-symbiotic interactions [25, 26], and recently it was shown that a miRNA (miR2111) traveling from the shoot to the root through the phloem controls the rhizobial infections of roots [27]. A large-scale profiling study on *Medicago* compared miRNA transcript abundances after AMF and rhizobial interactions and revealed a large diversity and plasticity of the miRNAome [28], and another study showed that 8 miRNA families were strongly altered in their expression during AM symbiosis in *M. truncatula* [29]. Investigations in tomato [30, 31] as well as on six Solanaceous species [32] identified a number of miRNAs and their potential targets known to be related to AMF and P nutrition. Furthermore, several studies showed miRNA expression profiles in various crops after different types of nutrient stress [33, 34]. The best studied miRNA families playing a role in P-starvation are miR399, targeting an ubiquitin-conjugating E2 enzyme [35]. miR399 was also reported to accumulate in arbuscules in *N. tabcacum* and *M. truncatula* [36]. MiR171h restricts AMF formation by targeting NSP2, a GRAS transcription factor [37–39], while miR171b positively regulates AM colonization [40]. MiR396 and miR393 have also been shown to regulate fungal colonization. Despite this progress on the role of miRNAs in regulating AMF root colonization, research on this highly complex signaling network is still in its infancy.

*N. attenuata*, an ecological model plant, native to the Sorthwestern USA has been well described in its interactions with herbivores. Not only many defensive compounds and the signaling events eventually leading to resistance, resilience and tolerance have been elucidated in this species (for review [41–43]), but the role of its small RNA machinery in herbivory has also been revealed. The genome of *N. attenuata* harbors three RDRs [44–46], four DCLs [47] and 11 AGOs [48]. RdR1, DCL3 and DCL4 and AGO8 were shown to contribute to *N. attenuata’s* herbivore resistance [44, 47, 49]. Furthermore, the context-dependent importance of AMF on *N. attenuata’s* growth and fitness has been shown. An RNAi line silenced in the expression of *CCaMK* (inverted repeat (ir)*CCaMK*) does not form a functional partnership with AMF [50, 51], as previously described for *Medicago* and *Lotus* [52, 53]. When these plants are competing with a fully AMF-functional isogenic line in the same pot, ir*CCaMK* plants have a fitness disadvantage and typical markers of P-starvation are expressed [54]. However, the composition and the role of miRNAs during *N. attenuata*-AMF interaction remain unknown. As the transcriptome and genome of *N. attenuata* have been published [55, 56], mapping of miRNAs and their targets is possible.

Here, we used the same experimental set-up as described in [54] of ir*CCaMK* plants competing with empty vector plants for a limited amount of nutrients in the same pot with and without AMF inoculum. We conducted a large-scale miRNome profiling to elucidate miRNAs important for root AMF colonization. We demonstrate a complex pattern of regulation with putative targets involved in defense, phytohormones and P-starvation. Of particular interest are miRNAs with a specific AMF-induced regulation of sequence variants.

## Methods

### Plant growth and sample collection

The same plant material as described in [54] was used. In brief, *N. attenuata* silenced in the expression of calcium and calmodulin dependent protein kinase due to an inverted repeat construct (ir*CCaMK*, line A09–1212–1, [50]) and empty vector (EV) plants were germinated on Gamborg B5 medium after surface sterilization with a 2% (*w*/*v*) aqueous solution of sodium dichloroisocyanuric acid (DCCS) and treatment with a smoke solution and gibberellic acid for 1 h [57]. 12 days after germination seedlings were transferred to 2 L pots filled either with living or autoclaved *Rhizophagus irregularis* inoculum (Biomyc Vital). Plants were paired, and each pot contained an EV seedling and an ir*CCaMK* seedling. During rosette stage plants were fertilized with 1/10 (50 μM) of the regular P concentration of hydroponics solution, and after elongation with ¼ (125 μM) of the regular P level. Six weeks after inoculation plants were harvested, the root systems of the plants carefully separated, washed in distilled water and immediately frozen in liquid nitrogen until further use.

### Library preparation for small RNA-Seq

Three biological replicates for each genotype and treatment were used for RNA extraction. Samples were ground with mortar and pestle and RNA extracted with a phase extraction method [58]. Purity, concentration and integrity of RNA samples were determined using Nanodrop and Agilent 2100 bioanalyzer in combination with RNA 6000n kit. Frozen RNA samples were shipped to the Max Planck Genome Center, Cologne for sequencing. Cluster generation and sequencing for each library was performed on Illumina HiSeq 2500 platform and 50 bp single-end reads were generated.

### Profiling of smRNome in *N. attenuata*- *arbuscular mycorrhiza* interactions

For each sample, the reads containing adapter and poly-N sequences were removed from the raw data. All the clean downstream analyses were based on clean data with high quality reads.

The clean reads were aligned to the *N. attenuata* genome [56, 59]. The genome aligned reads were further mapped to known mature miRNAs of 72 plant species obtained from miRBase v21. MiRDeep2 v2.0.0 [60] was used for alignment to the genome, identification of known miRNAs and prediction of novel miRNAs specific to *N. attenuata*-*AMF* interactions.

The identified known (conserved) or novel miRNAs for replicates were merged for each condition and reads present in at least two out of three replicates were retained for further analysis. Total counts were normalized using the Reads Per Million (RPM) method [61] and the median of merged miRNAs’ counts (i.e. RPM) was calculated.

The raw data of small RNA sequencing experiment have been deposited at NCBI and the accession number is PRJNA429556.

### Differential expression of miRNAs

To obtain the change in expression between the conditions, for both known and novel miRNAs, four pairwise comparisons were performed on the median RPM value for each miRNA. To estimate significantly differentially expressed miRNAs (DEmiRs), Chi-square [62] was performed and a corrected *p*-value < 0.05 after adjusting for multiple testing corrections using Benjamini-Hochberg method [63] was set as a cut-off.

### Target gene identification for miRNAs

To elucidate the biological processes that might be affected due to miRNAs, we identified target genes for DEmiRs. The bioinformatics method, psRNATarget ([64, 65] http://plantgrn.noble.org/psRNATarget/) was used for predicting the target genes using the default settings. The psRNATarget analysis server provides reverse complementary matching between miRNA and target transcript using a proven scoring schema, and target-site accessibility evaluation by calculating unpaired energy (UPE) required to ‘open’ secondary structure around miRNA’s target site on mRNA.

A gene ontology analysis of the predicted targets genes was performed using PANTHER Overrepresentation Test (http://pantherdb.org/tools/compareToRefList.jsp).

## Results

### Overview of small RNA profiles in AMF inoculated and non-inoculated roots of competitively grown EV and ir*CCaMK* plants

**Fig. 1:**
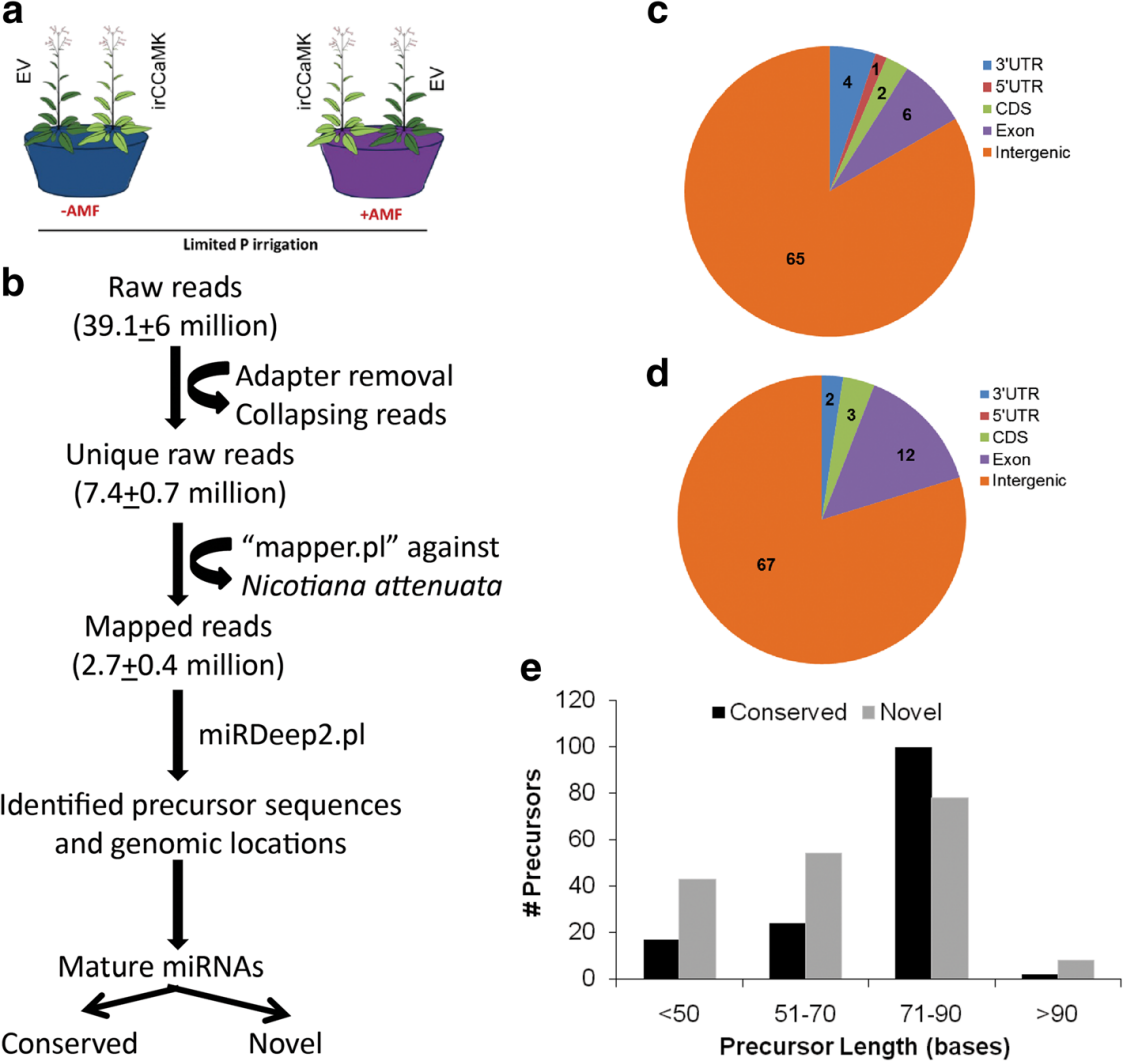
Elucidation of AMF-induced miRNA reprograming in *Nicotiana attenuata* roots. **a** Experimental set-up. Empty vector (EV) *Nicotiana attenuata* were competitively grown with an isogenic line silenced in the expression of a calcium and calmodulin protein kinase (ir*CCaMK*) with and without out arbuscular mycorrhiza (+/-AMF) inoculum. Three replicate samples for each of the four conditions were used for library preparation and small RNA-sequencing. **b** Pipeline adapted to identify miRNAs in EV and ir*CCaMK* plants with and without AMF inoculum. Counts for raw reads, unique reads, after removing adapter and collapsing, genome mapped reads, precursor sequences and genomic loci detected are provided at each step. All the filtering steps and their results are described in Material and Methods. **c**, **d** Genomic annotation for conserved and novel precursor miRNAs. Pie-charts show the distribution of precursor genomic loci of the *Nicotiana attenuata* genome for the conserved (**c**) and novel (**d**) miRNAs. The number of miRNAs originating from the 3’UTR, 5’UTR, coding sequences (CDS), exon and intergenic regions are shown. **e** Histograms show the size distribution of the precursor sequences for both, the conserved (black bars) and novel (grey) miRNAs. The x-axis represents the size of precursor sequences in bases and the y-axis shows number of unique precursor sequences

To characterize the smRNome in response to AMF we used a previously established set-up of competitively grown ir*CCaMK* plants, impaired in the interaction with arbuscular mycorrhiza and fully AMF-functional empty vector (EV) plants with and without *Rhizophagus irregularis* inoculum for the construction of libraries (Fig. 1a) [54]. Both genotypes were grown in the same pot without any barrier. Inoculated EV plants were well colonized and a accumulated transcripts of the AMF-specific P-transporters *NaPT4* and *NaPT5* in high abundance. In contrast, ir*CCaMK* plants while showing some AMF structures, and low transcript levels of the P-transporters, (Fig. 2 in [54]), which resulted from insufficient separation of the highly intertwined root systems during harvest. We sequenced 3 biological replicates per condition, and obtained 39.1 million raw reads. After adapter removal and filtering out the low quality sequences, the reads were mapped against the *N. attenuata* transcriptome resulting in 2.7 million reads (Fig. 1b). Using the miRDeep2 pipeline, we identified precursor sequences and genomic loci. We found 162 genomic loci, 78 conserved and 84 novel ones (Fig. 1c, d, Additional file 1: Table S1). More than three quarters of the sequences of conserved and novel miRNAs mapped to intergenic regions followed by exon sequences. Precursors with a length of 71–90 bases clearly dominated in abundance, in particular for conserved reads (Fig. 1e). The number of raw reads per sample was in a similar range for all samples, though non-inoculated ir*CCaMK* had the lowest number while inoculated plants of the same genotype had the highest number, but this difference was not reflected in the number of reads mapped to the genome (Table 1).

**Fig. 2:**
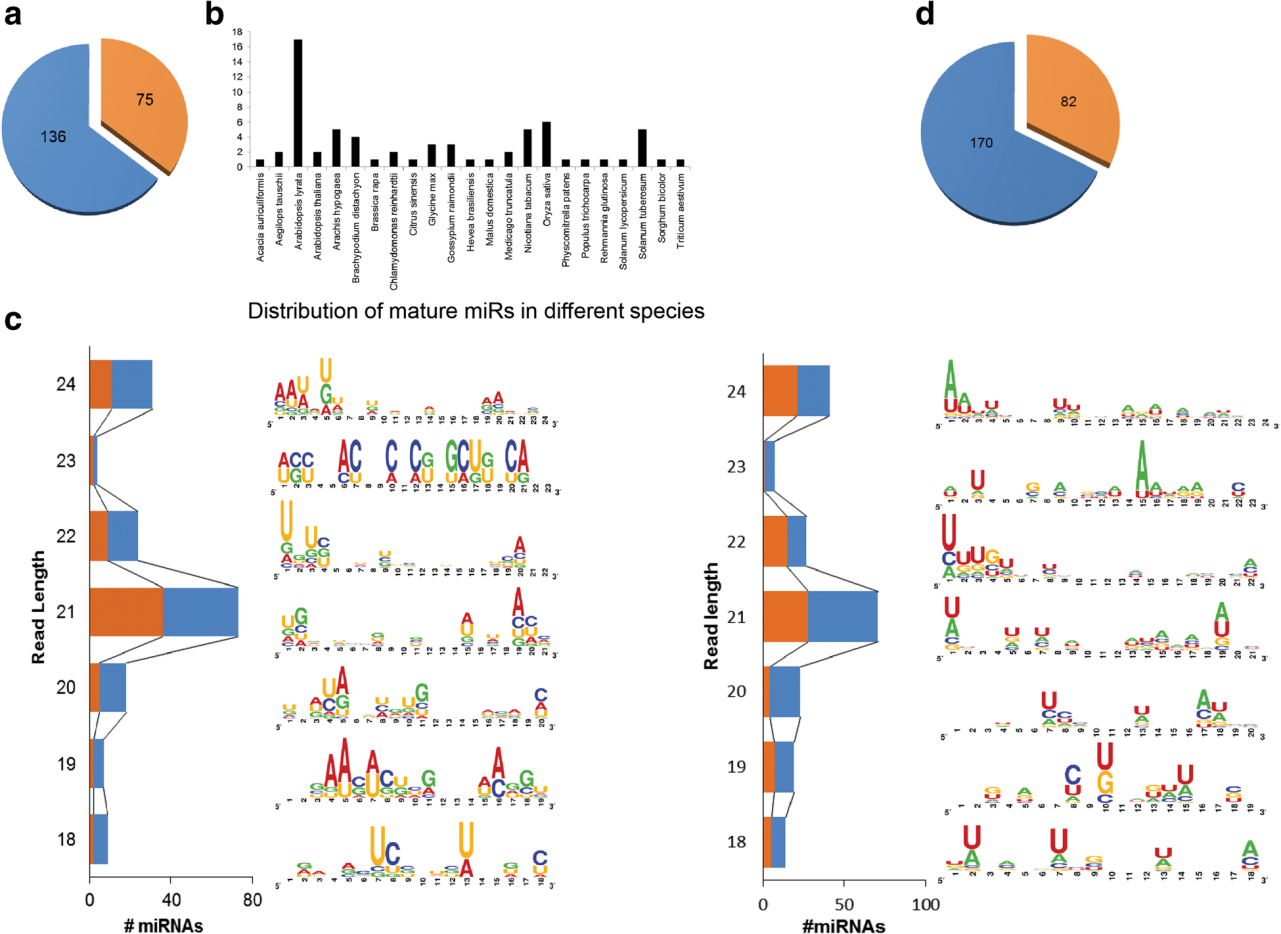
Features of conserved and novel miRNAs expressed in *Nicotiana attenuata* roots during AMF colonization in EV and ir*CCaMK* plants. **a** Two hundred and three conserved miRNAs (corresponding to 211 reads) were identified, 136 of which have been detected as miRNA-stars. **b** Analysis of distribution of conserved miRNAs reveals that the expressed Na-miRNAs were conserved in 23 plant species. The histogram shows the number of miRNAs conserved in each species. Sequence logos for conserved (**c**) and novel (**e**) miRNAs of different lengths. The majority of the sequences are 21 nt long for both conserved and novel miRNAs. The first nucleotide at 5′ is “U” for most of the miRNAs. **d** Two hundred and fifty two novel miRNAs were identified, 170 of these were detected as star sequences. Blue represents the star-mature miRNA sequences, orange denotes the consensus miRNA sequences

**Table 1:** Read statistics per sample. For the experimental set-up and labeling please see Fig. 1

Samples	#Raw Reads	#Unique Reads	Number of 18-30 nt Long Reads	#Reads mapped to Genome
EV + AMF1	47,099,031	7,068,199	5,671,699	2,507,142
EV + AMF2	38,926,386	7,403,723	5,953,754	2,557,302
EV + AMF3	45,382,090	7,045,154	5,700,915	2,365,720
EV-AMF1	34,654,264	7,603,801	5,859,834	2,858,917
EV-AMF2	30,929,486	6,140,793	5,070,138	2,685,363
EV-AMF3	41,461,357	7,703,684	6,519,373	3,538,388
irCCaMK+AMF1	45,452,011	8,722,024	6,096,462	2,364,341
irCCaMK+AMF2	42,322,161	8,064,104	5,863,175	2,236,918
irCCaMK+AMF3	43,479,739	8,107,352	6,223,535	2,660,752
irCCaMK-AMF1	31,354,853	7,041,399	5,567,009	2,850,628
irCCaMK-AMF2	30,414,054	6,608,922	5,314,658	2,799,453
irCCaMK-AMF3	37,553,383	7,633,436	6,119,711	3,205,640

### Profiles of conserved and novel miRNAs identified in *N. attenuata* roots

After further cleaning of the data, we identified 211 reads mapped to 67 conserved miRNAs (Additional file 1: Table S1), 136 of these reads were identified as miRNA stars (Fig. 2a). These conserved miRNAs were mapped against 72 plant species in miRBase and detected in 23 species (Additional file 2: Table S2) with the highest number of conserved miRNAs known from *Arabidopsis lyrata* followed by *Oryza sativa*, *N. tabacum* and *Solanum tuberosum* (Fig. 2b). Additionally, we found 252 reads predicted as novel miRNAs, of which 170 were detected as miRNA star sequences (Fig. 2d). The majority of the sequences (35.5% conserved, and 32.5% novel for mature miRNAs, and 64.4 and 67.5% for conserved and novel miRNAstars, respectively) were 21 nucleotides long followed by 24 nucleotides. For most of the miRNAs, the first nucleotide was “U”.

### Reprograming in abundances of conserved miRNAs depends on genotype and AMF inoculation

**Fig. 3:**
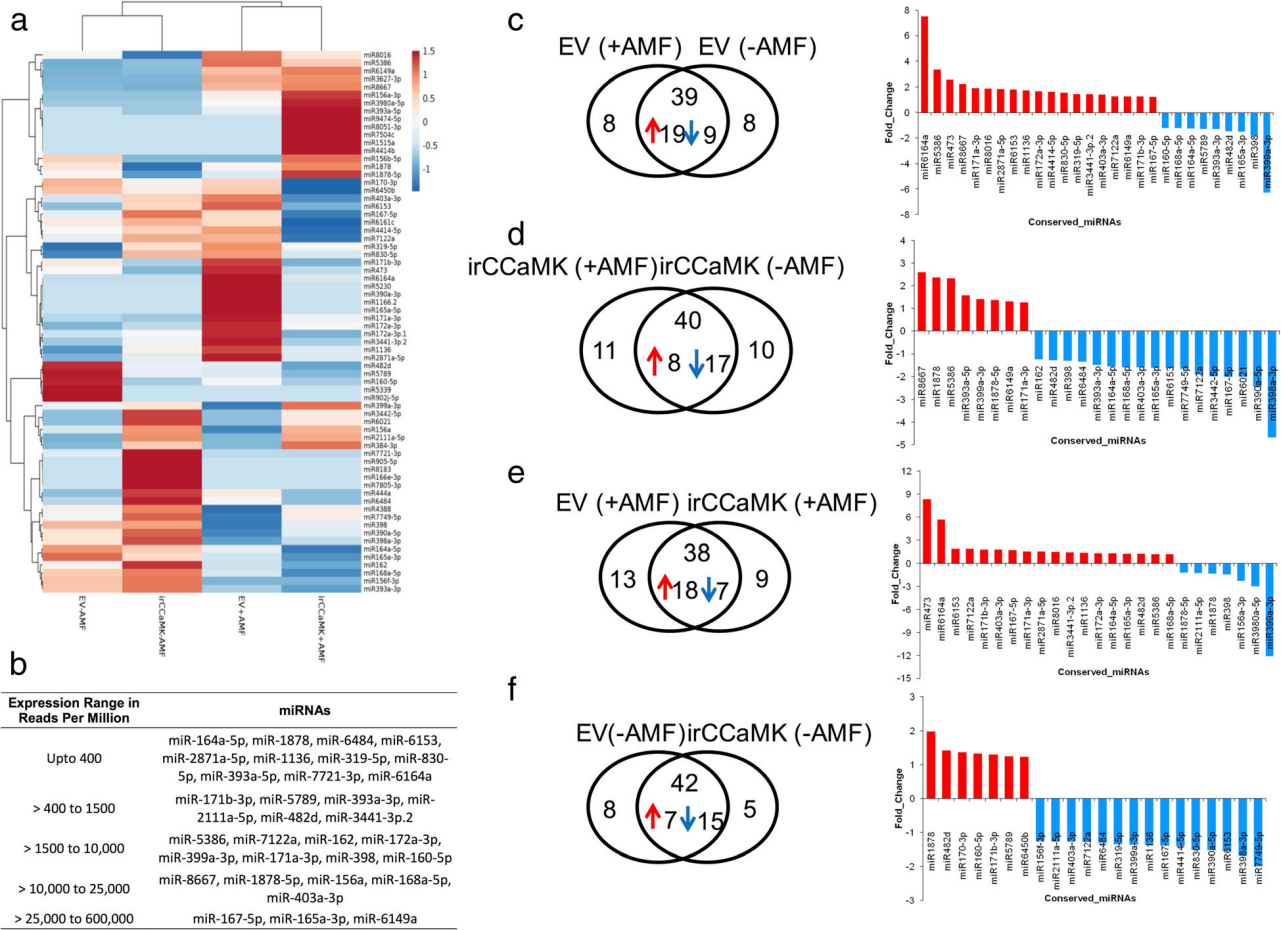
Differential abundance of conserved miRNAs in EV and ir*CCaMK* plants by AMF inoculation. **a** Unsupervised hierarchical clustering of normalized miRNA abundance values was performed using Pearson correlation and the average linkage method. Row values were centered and scaled using the unit variance scaling method. More abundant miRNAs are shown in red and less abundant ones are shown in blue. Differential accumulation of multiple miRNAs between the conditions was observed. **b** Na-miRNAs show a wide range of abundance patterns. The majority of the Na-miRNAs found in all four conditions accumulated less than 400 RPM (reads per million) values, whereas 8 miRNAs were highly abundant as they accumulated > 10,000 RPM. Six miRNAs accumulated 400–1500 RPM, whereas 8 miRNAs had RPM counts between 1500 and 10,000. The individual RPM values of each miRNA (in all four conditions) are provided in SI Table 1. **c**-**f** Differential accumulation of miRNAs using pairwise comparisons, computed at a *p*-value < 0.05 and fold-change > 1.2 or < − 1.2 cut-offs. The bar-charts for each comparison plot the up- and down-regulated differentially expressed (DE) miRNAs (miRs). The red color represents up- and blue denotes down-regulation

To explore differences in miRNA transcript abundances induced by AMF inoculation and genotype, we performed unsupervised hierarchical clustering on the normalized transcript abundances of the conserved miRNAs using a Pearson correlation and average linkage method. The two genotypes and treatments revealed a clearly distinct pattern of up- and down-regulation (Fig. 3a). Na-miRNAs show a wide range of abundance patterns. The majority of Na-miRNAs accumulated less than 400 RPM (reads per million), whereas 8 miRNAs were highly abundant as they accumulated up to 600,000 RPM (miR8667, miR1878-5p, miR156a, miR168a-5p, miR403-3p, miR 167-5p, miR165-3p, miR6149a). Six miRNAs accumulated 400–1500 RPM, and 8 miRNAs had RPM counts between 1500 and 10,000 rpm (Fig. 3b). A pairwise comparison of EV inoculated and non-inoculated plants using a *p*-value < 0.05 and fold-change > 1.2 or < − 1.2 cut-offs resulted in 28 differentially expressed miRNAs (DEmiRs). 19 of these were up- and 9 down-regulated (Fig. 3c, Additional file 3: Table S3). The most highly changed miRNAs were miR6164a, miR5366 and miR473 with higher abundances in inoculated EV plants, while miR390-5p and miR399a-3p were far less abundant compared to non-inoculated plants of the same genotype (Fig. 3c, Additional file 3: Table S3). Furthermore, 8 miRNAs were specific to each condition. For the comparison between inoculated and non-inoculated ir*CCaMK* plants, out of 61 miRNAs, 40 were expressed in both conditions and 25 were DEmiRs. The strongest differences in abundance were observed for miR8667 (up) and miR399a-3p (down) (Fig. 3d, Additional file 3: Table S3). Furthermore, for the comparison of the two inoculated genotypes 25 miRNAs were differentially expressed, of the 38 commonly expressed miRNAs (Fig. 3e, Additional file 3: Table S3). Here again, as shown for the comparison of inoculated and non-inoculated EV plants, the strongest changes were observed for miR473, miR6164a (up) and miR399s-3p (down). For the comparison of ir*CCaMK* (-AMF) versus EV (-AMF) 22 DEmiRs (of 42 miRNAs in total) were obtained (Fig. 3f). The fold change for all DEmiRs in this comparison was lower than for the other comparison and reached a maximum of 2, indicating that differences in abundances of conserved miRNAs between the two genotypes without inoculum were minor. As a proof of concept, we validated the expression of 4 miRNAs in an independent experiment and the results corroborated with those presented here (data not shown).

### Changes in abundances of novel miRNAs depending on genotype and AMF inoculation

**Fig. 4:**
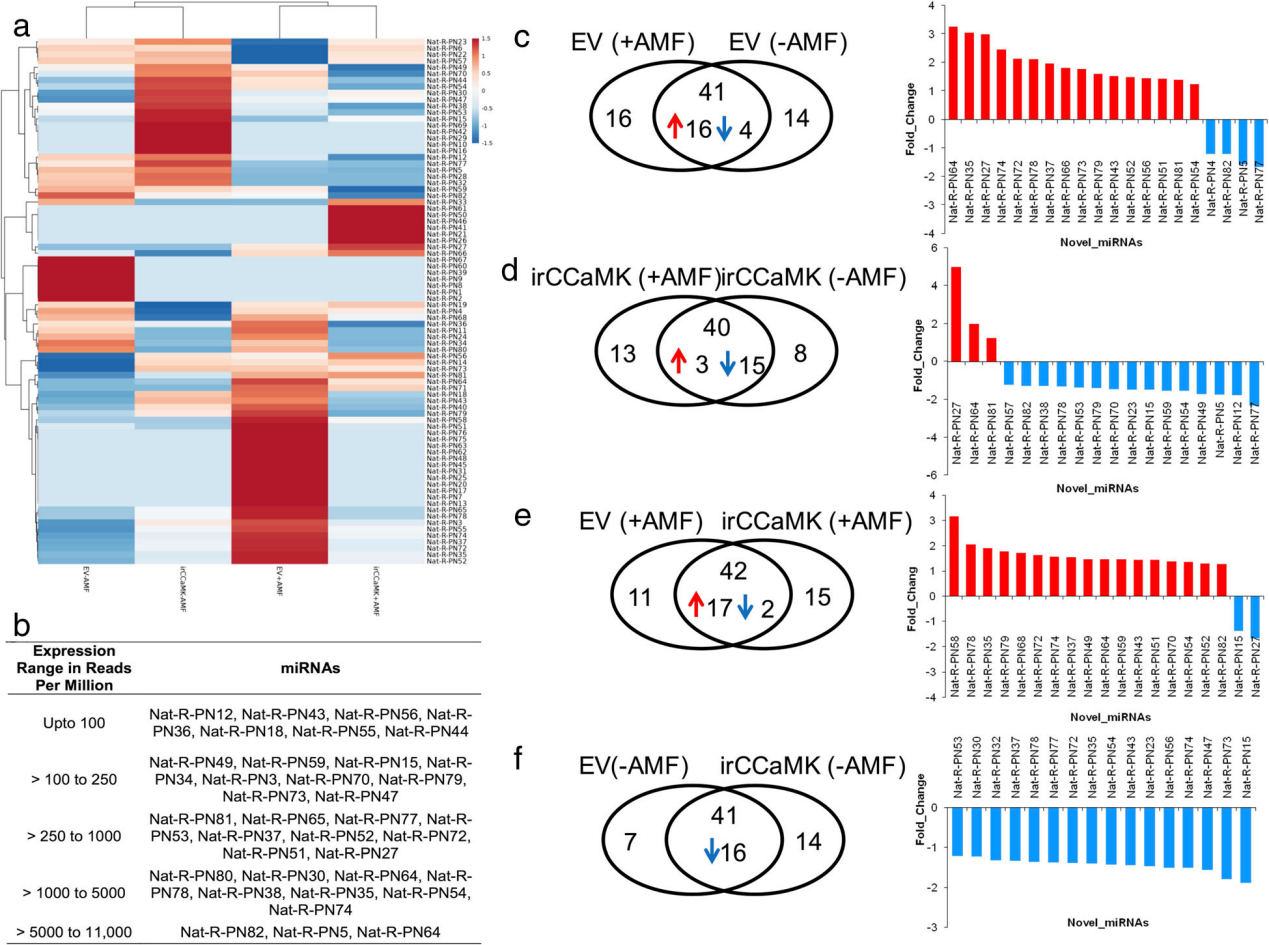
Investigation of reprograming of abundance of novel miRNAs in EV and ir*CCaMK* plants by AMF inoculation. **a** Unsupervised hierarchical clustering of normalized miRNA abundance values was performed using the same methods as for the conserved miRNAs. The heatmap shows large number of novel miRNAs are enriched in the presence of AMF in EV (as shown by the bottom red color block). Similarly, a large number of novel miRNA reads were over-expressed in the absence of AMF in ir*CCaMK* (-AMF) plants as shown in the upper red colored block in the heatmap. **b** Expression range of novel miRNAs in all the four conditions. Overall novel miRNAs have lower abundance values as compared to the conserved ones. The individual RPM values of each miRNA (in all four conditions) are provided in SI Table 1. **c**-**f** Differential expression of novel miRNAs in the pairwise comparisons of four conditions computed at a p-value < 0.05 and fold-change cut-off of > 1.2 or < − 1.2. The bar-charts for each comparison plot the up- and down-regulated differentially expressed miRNAs (DE-miRs). Red color represents up- and blue denotes down-regulation

We also analyzed novel miRNAs in the same way as we had done with the conserved miRNAs, and again found a clustering into AMF inoculated and non-inoculated plants (Fig. 4a). The heatmap further revealed that a large number of novel miRNAs are more abundant in inoculated EV plants compared to the other treatments/genotypes. Interestingly, a large number of novel miRNA reads was more abundant in the absence of AMF in ir*CCaMK* plants (Fig. 4a). The number of reads for novel miRNAs was overall lower than for conserved miRNAs in all four conditions, and maximum values reached less than 11,000 RPM, in contrast to more than 25,000 RPM for conserved miRNAs (Fig. 4b). Highest abundances were observed for Nat-AMF-PN82, Nat-AMF-PN5, Nat-AMF-PN64.

The pairwise comparisons using a *p*-value < 0.05 and fold-change cut-off of > 1.2 or < − 1.2 also resulted in a different pattern. The comparison of novel miRNAs for the effect of AM inoculation in EV resulted in 20 DE-miRs, 16 were up- and only 4 were down-regulated in EV inoculated versus non-inoculated plants (Fig. 4c, Additional file 3: Table S3). The opposite pattern – only three DEmiRs were upregulated and 15 downregulated - was observed for the effect of AMF inoculation on ir*CCaMK* plants (Fig. 4d, Additional file 3: Table S3). The comparison of the two genotypes with AMF treatment showed a similar pattern as the treatment effect in EV, and many significantly enriched miRNAs were the same between the two comparisons (Fig. 4e). As already shown for conserved miRNAs, this finding indicates that non-inoculated EV plants and inoculated plants impaired in the interaction with AMF led to a similar induction of novel miRNAs compared to AMF colonized EV plants. For the comparison of non-inoculated ir*CCaMK* and EV plants, all 16 DE-miRs were less abundant in non-inoculated ir*CCaMK* plants (Fig. 4f, Additional file 3: Table S3).

**Fig. 5:**
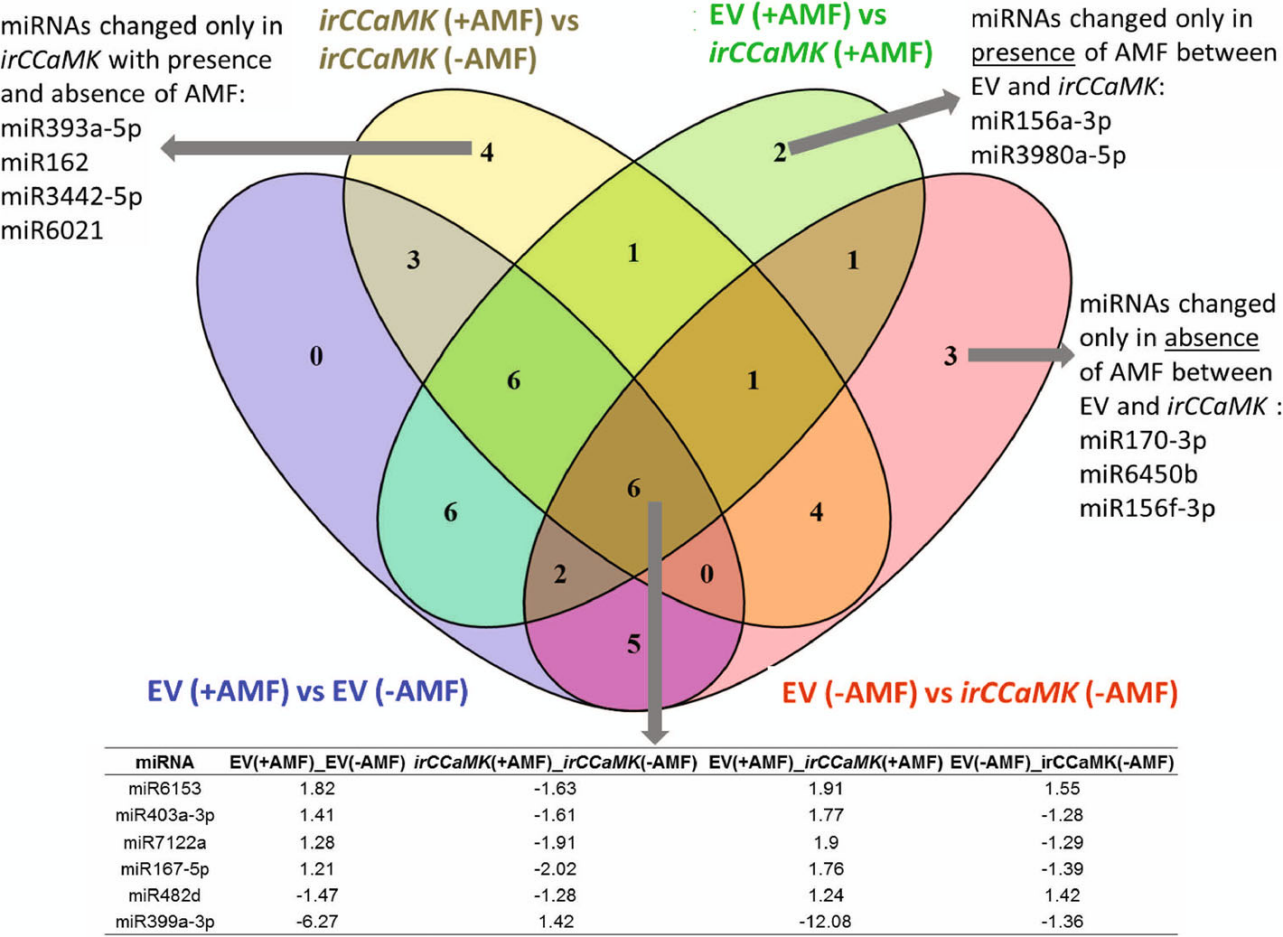
Comparing conserved differentially expressed miRs in the four conditions demonstrates complex patterns of miRNA accumulation. The Venn diagram shows miRNAs that were changed specifically with or without AMF or in a specific genotype (EV or ir*CCaMK*) or in both

We also compared the enriched DE-miRs in the four conditions, confirming the complex patterns of miRNA accumulation. The Venn diagram shows miRNAs that were changed specifically in one condition or in a specific genotype (EV or ir*CCaMK*) (Fig. 5). Four miRNAs were significantly altered when comparing AMF inoculated and non-inoculated ir*CCaMK* plants, namely miR393a-5p, miR162, miR3442-5p, miR6021. Two miRNAs were specifically changed only due to AMF inoculation in EV plants (miR156a-3p and miR3980a-5p). Six miRNAs were differentially changed in both, EV or *irCCaMK* plants, with and without AMF inoculum (Fig. 5). Interestingly 5 of these miRNAs were less abundant in inoculated ir*CCaMK* plants versus non-inoculated plants of the same genotype, while miR399a-5p was strongly enriched. Comparisons between inoculated EV plants and non-inoculated EV plants and vs inoculated ir*CCaMK* plants showed the opposite pattern – miR6153, miR403a-3p, miR7122a and miR1675p accumulated, while miR399a-3p was less abundant. Hence, these six miRNAs are directly or indirectly linked to AMF inoculation.

### Sequence variants of conserved miRNAs show distinct treatment and genotype effects

**Fig. 6:**
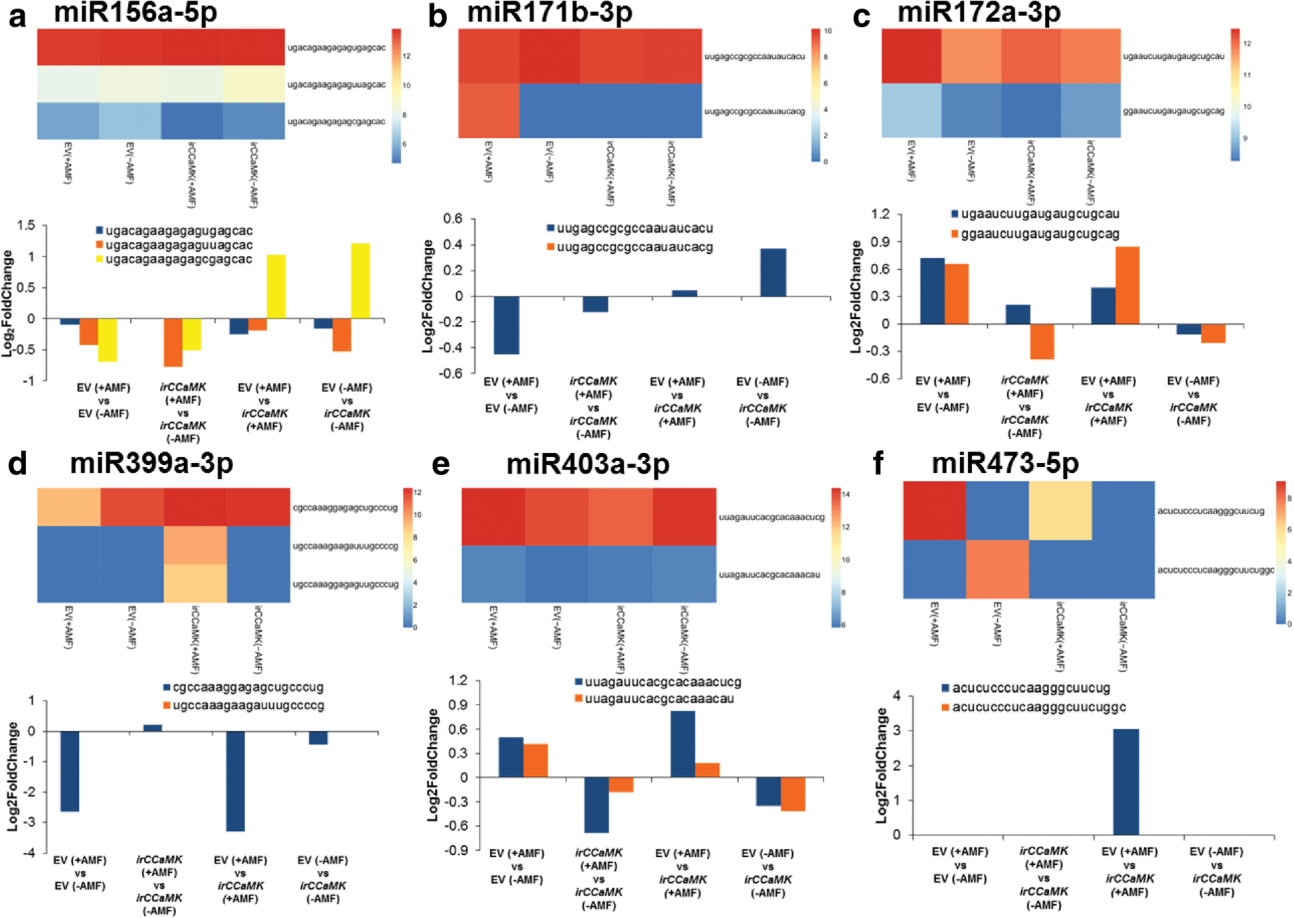
Sequence variants observed for conserved miRNAs in EV and ir*CCaMK* plants demonstrate strong differences in abundance depending on the genotype and AMF inoculation. Each miRNA is depicted by two panels, a heatmap showing the normalized RPM values for each sequence variant in the four conditions, and a bar-chart representing the differential abundances of these sequence variants in the four comparisons (**a**-**f**). Three sequences with one nucleotide difference towards the 3′ end were observed for miR156a-5p (**a**). These variants have originated from different genomic locations with different precursor sequences. One of the variants was highly abundant (> 9000) in all four conditions (blue color in heatmap), whereas the other sequence variants were far less abundant (< 100 RPM values). For miR171b-3p (**b**) two sequence variants were found which originated from different genomic loci. One of the variants was only captured in EV (+AMF) with > 400 RPM values. For miR172a-3p (**c**) 2 sequence variants were found, both originating from different genomic loci. All three sequence variants of miR399a-3p (**d**) were captured in ir*CCaMK* (+AMF), while only one sequence variant was found in EV and non-inoculated plants. MiR403a-3p (**e**) and miR473-5p (**f**) were sequenced with two different sequences, originating from the same chromosome

MiRNAs may be heterogeneously transcribed and may be expressed in a cell- or condition-specific manner [66]. Therefore, we further investigated sequence variants of the conserved miRNAs and their expression in each condition. All the miRNAs that accumulated an isoMiR are described in Fig. 6. Three sequences with one nucleotide difference towards the 3′ end were observed for miR156a-5p (Fig. 6a). These variants originated from different genomic locations with different precursor sequences. One variant accumulated more than 9000 RPM in all four conditions, whereas the other sequence variants were far less abundant (< 100 RPM). The third sequence variant was specifically enriched in EV compared to ir*CCaMK* independent of inoculation. For miR171b-3p (Fig. 6b), two sequence variants were found which originated from different genomic loci. One of the variants was only captured in AMF colonized EV (+AMF) roots with more than 400 RPM, and seems to be directly related to a functional symbiosis; the sequence is almost identical with miR171g from tomato [31] and similar to miR171h from *Medicago* [37], indicating a strong conservation across different species. The other was less abundant after AMF colonization in both genotypes (Fig. 6b). For miR172a-3p, two sequence variants were found; both originating from different genomic loci (Fig. 6c). miR172 has been shown to play an important role in the early events during nodule formation [67]. The two variants accumulated in AMF-colonized EV plants compared to non-colonized EV plants and plants impaired in the interaction with AMF; from these patterns, we infer that they are involved in the regulation of AMF root colonization.

All three sequence variants of miR399a-3p were captured in ir*CCaMK* (+AMF), while only one sequence variant was found in EV and non-inoculated plants. This sequence variant was far less abundant in inoculated EV plants compared to non-inoculated plants and inoculated ir*CCaMK* plants (Fig. 6d); hence, the accumulation of miR399a-3p is highly specific for inoculated ir*CCaMK* plants competing with EV plants for the same limited amount of P. MiR399 is well described to be induced by P-starvation, consistent with the highly conserved role of miR399 in P-deficiency. MiR403a-3p (Fig. 6e) and miR473-5p (Fig. 6f) were sequenced with two different sequences, originating from the same chromosome. MiR403a-3p sequence variants are 10 kilobases apart on the genome and transcript accumulation was higher in EV (AMF+) plants for both variants. The two sequence variants for 473-5p originated from the same location with different precursor sequences. Interestingly, the first sequence of 473-5p was captured in AMF inoculated plants independent of the genotype, but to a lesser extent in ir*CCaMK* (AMF+) than in EV, while the second one was only captured in non-inoculated EV (-AMF) plants.

In summary, these findings show that the abundance of sequence variants of miRNAs are specifically altered in close association with a functional symbiosis and AMF root colonization.

### Putative targets of selected miRNAs include genes related to P-starvation, phytohormone signaling and defense

**Fig. 7:**
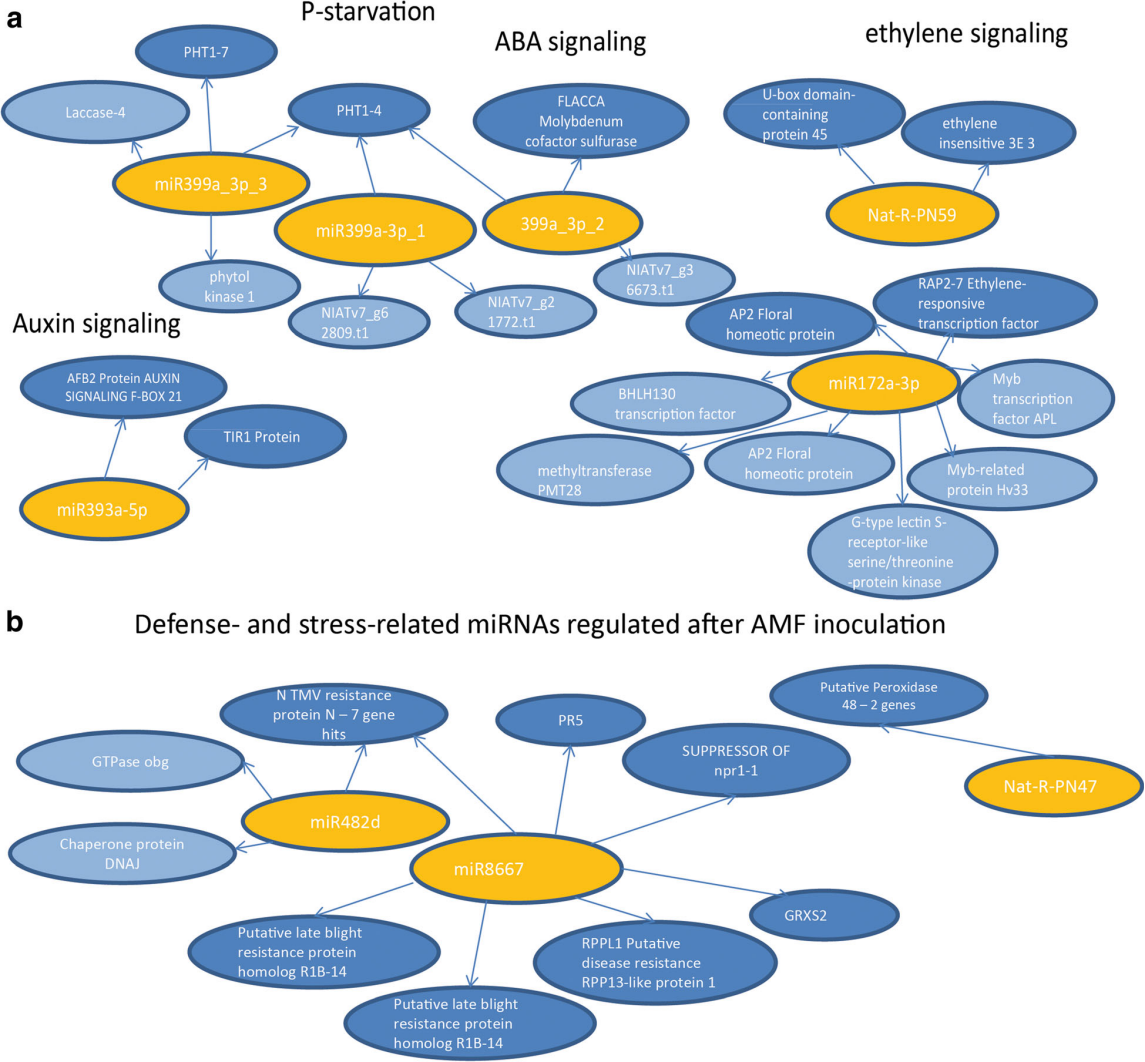
Selected miRNAs significantly changed by AMF inoculation or genotype and their putative targets. **a** miRNAs and their putative target genes involved in P-starvation and phytohormone signaling. **b** miRNAs and their putative target genes involved in pathogen defense and stress. Circles shown in dark blue are putative targets related to the functions mentioned in the title, further putative targets are depicted in light blue

We predicted putative targets of selected miRNAs against the transcriptome of *N. attenuata* for 30 conserved and novel DEmiRNAs using psRNATarget tool [64, 65] (Additional file 4: Table S4). We selected miRNAs based on their expression pattern (significant up/down-regulation due to treatment or genotype) and their miRNA isoform expression pattern. Except for two miRNAs (miR5386, Nat-R-PN5), all selected miRNAs had at least two putative targets (Additional file 4: Table S4). A gene ontology analysis of the predicted targets revealed a strong enrichment in genes related to metabolic and cellular processes – terms which are related to development and nutrient supply – as well as responses to stimulus and stress (Additional file 5: Table S5). Interestingly, there is also a significant enrichment of genes related to reproduction. Further putative targets of selected miRNAs are involved in pathogen defense and stress, and in phytohormone metabolism. To better illustrate the regulatory network, we depicted some miRNAs and their putative targets (Fig. 7). We selected miRNAs whose targets are described in literature to play a role in plant signaling or in response to AMF colonization. In accordance with the literature, miR399 targeted genes, such as phytolkinase and the phosphate transporters PHT1–4 and 1–7, which are involved in the P starvation response. The expression pattern of miR399 – namely, strong upregulation in plants impaired in AMF colonization (ir*CCaMK*) competing with AMF-colonized plants (EV) and in non-inoculated compared to inoculated EV plants – is consistent with the P-starvation of ir*CCaMK* compared to EV plants in the presence of AMF [54]. The three sequence variants were found to target the same putative target PHT1–4. Conserved miR393 targets the auxin-related F-Box proteins TIR1 and AFB2. The novel miRNA Nat-R-PN59 as well as miR172a-3p have two transcription factors related to the ethylene response pathway as putative targets (EIN3, RAP2, Fig. 7a). Ethylene has been shown earlier to be an important factor in AMF colonization [68].

We also observed that the putative targets of several miRNAs that are particularly enriched in AMF inoculated plants - are involved in defense (Fig. 7b). MiR8667 and miR482 are assumed to regulate NBS-LRR proteins, which are very well described in pathogen defense; though here they show the opposite patterns – while miR8667 is strongly enriched in AM colonized EV plants, miR482 is depleted. Furthermore, miR6153 putatively targets cysteine proteinase which is also associated with defense and it shows highest transcript abundance in inoculated EV plants.

Several GRAS transcription factors have been described to play a key role in a successful AMF symbiosis [69, 70], and some are known to be regulated by miRNAs [69]. Our target analysis also revealed a number of GRAS transcription factors as putative targets. Particularly striking was that NIATv7_g16119, similar to nodulation signaling protein 2, was identified as a putative target of miR171b-3p_1, known from *Medicago* to be important for AMF colonization [40, 71]. A phylogenetic analysis of NIATv7_g16119 revealed orthologues in other species including *Medicago*, *Lotus* and tomato, a result consistent with the notion that the putative NSP2 found in our study is closely related to the NSP2 from legumes (Additional file 6: Figure S1). Additional targets of miR171b-3p in our study were scarescrow-like proteins (Additional file 4: Table S4), a finding which is also consistent with previous studies [31, 72]. The phylogenetic analysis strongly supports that the regulation of the AMF symbiosis is highly conserved across species.

## Discussion

While the regulation of AMF by miRNAs has been characterized for a few specific miRNAs, and global miRNA profiles have been characterized for both tomato and Medicago in response to AMF, there is little overlap in the results, and many regulatory elements remain to be discovered. In a previous study we showed that *N. attenuata* plants that were impaired in the interaction with AMF (ir*CCaMK*) suffered from P-starvation and reduced growth when competing in shared bots with a fully AMF-functional isogenic line (EV) for the same limited amount of nutrients when inoculated with AMF [54]. Here we used the same experimental set-up and characterize the miRNA profiles of *N. attenuata* roots to better understand the role of smRNAs in this interaction. We found major changes in the miRNA profiles due to AMF colonization, with clearly distinct patterns for a fully functional interaction with the fungal partner (EV plants) compared to inoculated plants impaired in the interaction (ir*CCaMK*) and non-inoculated plants. AMF inoculation and CCaMK signaling have a major effect on miRNA regulatory networks. Modulation of conserved miRNAs and their known functions reflected AMF status.

A comparison of the two treatments (with and without inoculum) and the two genotypes (EV, ir*CCaMK*) revealed a distinction in inoculated and non-inoculated plants. 44 conserved DEmiRs were obtained in the four comparisons. Of these, six miRNAs differentially accumulated in all four comparisons (Fig. 5). These six are highly different in their abundance and they have been shown previously to be defense- and nutrition-responsive in other species.

Our experimental set-up using non-inoculated plants but also a line impaired in the interaction with AMF which contained a minor contamination of EV roots [54] allowed us to find miRNAs specifically induced by AMF. DEmiRNAs with different sequence variants (Fig. 6) revealed the regulation of some miRNAs only in inoculated EV plants, while others also accumulated in inoculated ir*CCaMK* roots, but to a lesser extent. Some of the conserved miRNA families differentially enriched after AMF treatment are known to be involved in symbiotic interactions such as miR156, miR171, miR393 and miR482 [73] (Additional file 7: Table S6). However, in contrast to *Medicago* treated with mycorrhizal liopchitooligosaccharides [28], two variants of miR156 were down-regulated in AMF inoculated roots compared to non-inoculated EV and to inoculated ir*CCaMK* plants. The known targets for miR156, the SQUAMOSA promotor binding like transcription factors [74] which are involved in the regulation of root development [75], were also found in this analysis as putative targets. Additionally, this multi-miRNA family may target pentatricopeptide repeat-containing protein and serine/threonine protein phosphatase 2A in *N. attenuata*. Based on the contrasting results for *Medicago* and *N. attenuata,* we infer that miR156 only plays an indirect role in AMF inoculation and might be more related to the nutritional status or development in our system. This inference is supported by other studies indicating a role of miR156 in the P starvation response [32, 76] and the vegetative phase change [77].

The role of some members of the miR171 family has been elucidated in detail; miR171h was shown to limit symbiotic root colonization [39], while miR171b stimulates root colonization by regulating other miR171 family members [40]. An upregulation of miR171 family members in symbiotic interactions was consistently observed in several plant species including tomato and other Solanaceous species [30–32]. We found a specific accumulation of one of the sequence variants of miR171 in AMF inoculated EV roots. The expression pattern of one of sequence variants of miR473 reflects the pattern of AMF-specific P-transporter expression [54] and is an interesting candidate for further investigations. Putative targets of miR171 are NSP2 as found in *M. truncatula*, and scarecrow-like protein 6 and 9. All three putative targets belong to the GRAS transcription factor family. Many members of this family have been shown to be specifically regulated by AMF [69]. NSP2 and miR171h expression show a negative correlation in AMF-inoculated *Medicago* plants, and it is assumed that their expression is tightly correlated with the nutritional status of the plant [39]. Furthermore, NSP2 is required for AMF-associated LCO signaling, and DELLAs interact with NSP2 (see below) [78]. A phylogenetic analysis revealed a high similarity of NSP2 from *Medicago* with the putative NSP2 protein found here, suggesting that this part of the plant-AMF signaling is conserved across species.

Similarly, miR473 was specifically induced by AMF, though in contrast to miR171, one of the sequence variants of miR473 strongly accumulated in AMF-inoculated EV plants, and to a far lesser extent in inoculated ir*CCaMK* plants (Fig. 6). One of the putative targets of miR473 is a DELLA protein, another type of GRAS transcription factors (gibberellic acid insensitive - GAI). DELLA proteins have been shown to be negative regulators of gibberellic acid (GA) signaling, and their presence is required for arbuscule formation in *Medicago* [79]. DELLA proteins interact with NSP2 and NSP1 [78]. The DELLA protein found as putative target in the present study differs from the two DELLA proteins known from *Medicago*, and only shows 34 and 35% identity with MtDella1 and MtDella2 at the protein level, while the *N. attenuata* genome contains four other DELLA proteins with higher similarity to these two *Medicago* DELLAs than the one found here. Furthermore, miR473 has three other putative targets: a medium-chain-fatty-acid--CoA ligase, a plastid-lipid-associated protein and protein SEC13 homologue (Additional file 4: Table S4). The first being a component of lipid metabolism, the second is thought to be involved in lipid transport across the membrane and the third is also thought to be involved in transport processes. The specific upregulation of one of the isoforms of miR473 may be related to lipid transfer between plant and AMF [4, 5].

We found two miR399 sequence variants which only accumulated in inoculated ir*CCaMK* plants competing with EV plants, and a third variant was strongly enriched in ir*CCaMK* (+AMF) plants, but was also expressed to a lesser extent in EV, independent of treatment. The miR399 family is highly conserved across species and well described to be enriched in response to P-starvation [34, 80], but miR399 was also shown to be specifically induced in arbuscules [36] and in AMF-inoculated tomato roots and leaves [30]. As previously shown, ir*CCaMK* competing with fully functional EV plants in the presence of AMF plants suffer from P – starvation, while there is only a minor effect on P-status without inoculum [54]. The patterns of miR399 accumulation in ir*CCaMK* plants were consistent with these previous findings based on P-analyses and gene expression studies.. The putative targets of miR399 found for *N. attenuata* are members of the PHT1 family phosphate transporters, phytolkinase, IRX12 laccase-4 and a FLACCA molybdenum cofactor sulfurase. We had analyzed the expression of PHT1 family transporters with the same set-up in a previous study, and found that PHT1 family transporters were up-regulated in leaves but not in roots in inoculated ir*CCaMK* plants compared to EV (see Fig. 5, [54]). So far, PHT1 family transporters have not been experimentally demonstrated to be targets of miR399, but are rather indirectly regulated via PHO2 [34]. PHO2 has been described to be down-regulated by a high miR399 expression [34, 81]. This is consistent with our results and the previously published qPCR analyses demonstrating a weak expression of PHO2 in inoculated ir*CCaMK* plants compared to EV competing for the same amount of nutrients (Fig. 5 in [54]). The additional putative target of miR399 in *N. attenuata*, phytolkinase, is not a classical marker of P-starvation, but may be linked to P-deficiency via altered plasma membrane lipid metabolism that is commonly associated with P-deficiency [82, 83]. *Laccase4* has also been implicated in several types of nutrient stress (carbon, nitrogen, sulfur deficiency), though in Arabidopsis LAC4 was regulated by miR397b [33].

Interestingly, we also found a differential accumulation of miR393 which regulates auxin translocation by targeting the F-box receptors TIR, AFB2 and AFB3. MiRNA393 is down-regulated after AMF inoculation in rice and tomato [30, 84], and the overexpression of miRNA393 in these two species inhibits the formation of arbuscules [84]. Furthermore, miR393 represses the expression of the above-mentioned F-box receptors leading to the restriction of a bacterial plant pathogen due to repression of auxin signaling. However, in the present study, the sequence variant targeting the F-Box proteins is barely regulated, while the second sequence – which is significantly less expressed in AMF inoculated plants compared to non-inoculated plants and to AMF colonized ir*CCaMK* plants – has other putative targets; thus the role of miR393 family members in *N. attenuata* needs further investigation.

In addition to auxin, elements of the ethylene signaling pathway are putative targets of the conserved miR172 and of the novel Nat-R-PN59. In accordance with targets described in other species, miR172 targets APETALA2 and ethylene-responsive transcription factors. Their role has mainly been shown for flower development, flower timing and growth and development [85], but they are also altered by biotic stress and known from lipid metabolism [86]. Additionally, miR172 controls the levels of nodulation by regulating AP2 transcription factor in soybean [67] and in common bean [87], and there is an interplay between miR156 and miR172 [67]. The specific enrichment of miR172 in AMF-inoculated EV plants indicates that this miRNA may stimulate AMF root colonization by repressing AP2 expression, similar to the effect observed during nodulation.

Other putative targets of DE-miRNAs are involved in plant defense against pests and pathogens. In particular, miR8667 is strongly enriched in AMF inoculated EV and ir*CCaMK* roots, while miR482 is significantly more abundant in AMF inoculated EV plants compared to ir*CCaMK*, but the highest levels were observed in non-inoculated EV roots. One of the putative target of both miRNAs are TMV resistance protein N, which belong to the TIR-NBS (nucleotide binding site)-LRR (leucine rich-repeat) class of plant resistance proteins, a large gene family in the plant genome [88]. The constitutive upregulation of plant defenses in the absence of pathogens is costly [89] and selected for regulatory mechanisms that elicit expression on demand. miR482 has been shown to silence NBS-LRR disease resistance genes in tomato; upon pathogen infection the miR482 machinery is suppressed leading to an enhanced production of pathogen-inducible expression of NBS-LRR proteins and increased resistance [90]. The enrichment of miR8667 in the present study may avoid the upregulation of specific NBS-LRRs to enable the hosting of mycorrhizal fungi, while miR482 may target other another TMV resistance N-like proteins of this large multigene family leading to increased pathogen resistance.

## Conclusions

In conclusion, we found a complex pattern of miRNA expression, a number of the DEmiRNAs showing specific enrichment or depletion only in plants capable of a fully functional interaction with AMF. Putative targets of specifically regulated miRNAs are involved in phytohormone metabolism and plant defense. The study provides a rich foundation for future detailed functional analysis of specific miRNAs. These next analyses include (1) determining the molecular mechanism of action of miRNA-mediated regulatory network in AMF symbioses to investigate which miRNAs are specifically dependent on AMF and which are more related to conseqences of the interaction, such as specific growth conditions and nutritional status, (2) deciphering the role of *CCaMK* in regulating miRNA expression, and (3) elucidating the molecular players of the smRNA pathway such as AGOs, involved in AMF mediated regulation. *N. attenuata* offers a unique system in which to examine the functional specialization of the smRNA machinery, which has been demonstrated for herbivory-related responses.

## Additional files

Additional file 1: **Table S1.** Conserved and predicted novel miRNAs identified in the four sample types and log fold change for pairwise comparisons. (XLSX 72 kb)

Additional file 2: **Table S2.** Mature miRNAs conserved to other species when mapped against 72 plant species in miRBase. (XLSX 12 kb)

Additional file 3: **Table S3.** Differentially expressed miRNAs for EV and ir*CCamK* with and without AMF. (XLSX 41 kb)

Additional file 4: **Table S4.** Target genes for selected DEmiRs using psRNA Target and target description. (XLSX 667 kb)

Additional file 5: **Table S5.** Gene Ontology analysis of putative targets of differentially enriched miRNAs. (XLSX 11 kb)

Additional file 6: **Figure S1.** Phylogenetic analysis of NIATv7_g16119, similar to nodulation signaling protein 2 in *Medicago* (both in red), a putative target of miR171b-3p_1, compared to orthologues in different plant species [69] and to another GRAS transcription factor (AMVG91704.1, RAM1) known from *Lotus japonicus* to be important for AMF colonization [18]. The tree was constructed with Genious tree builder using the Jukes-Cantor genetic distance model and the neighbor-joining tree builder based on the amino acid sequences. TOBAC – *Nicotiana tabacum*, NICSY – *Nicotiana sylvestris*, SOLTU – *Solanum tuberosum*, NICAT – *Nicotiana attenuata*, ARATH – *Arabidopsis thaliana*, POPTR – *Populus trichocarpa*, ORYSJ- *Oryza sativa*, GOSHI – *Gossypium hirsutum*, MEDTR – *Medicago truncatula*, PEA – *Pisum sativum*, CAJCA – *Cajanus cajan*, RICCO – *Ricinus communis*, CAPAN – *Capsicum annuum*, SOLCH – *Solanum chacoense,* SOLLC - *Solanum lycopersicum*, CUCUME – *Cucumis sativus*, ARALL – *Arabidopsis lyrata*, MARPO – *Marchantia polymorpha*, (PDF 1754 kb)

Additional file 7: **Table S6.** miRNAs differentially expressed in this study with (+) and without (−) AMF inoculum in the two genotypes (empty vector [EV] and irCCaMK) and their expression and function described in literature. (DOCX 109 kb)

## Abbreviations

AMF: Arbuscular mycorrhizal fungi
CCaMK: Calcium- and calmodulin-dependent protein kinase
DEmiR: Differentially expressed micro RNA
EV: Empty vector
ir: Inverted repeat
miRNA: Micro RNA
