# Supplementary figures and images for: Complex regulation of microRNAs in roots of competitively-grown isogenic Nicotiana attenuata plants with different capacities to interact with arbuscular mycorrhizal fungi
Source: BMC Genomics. 2018 Dec 17;19:937. doi: 10.1186/s12864-018-5338-x (PMC6296096; doi:10.1186/s12864-018-5338-x)

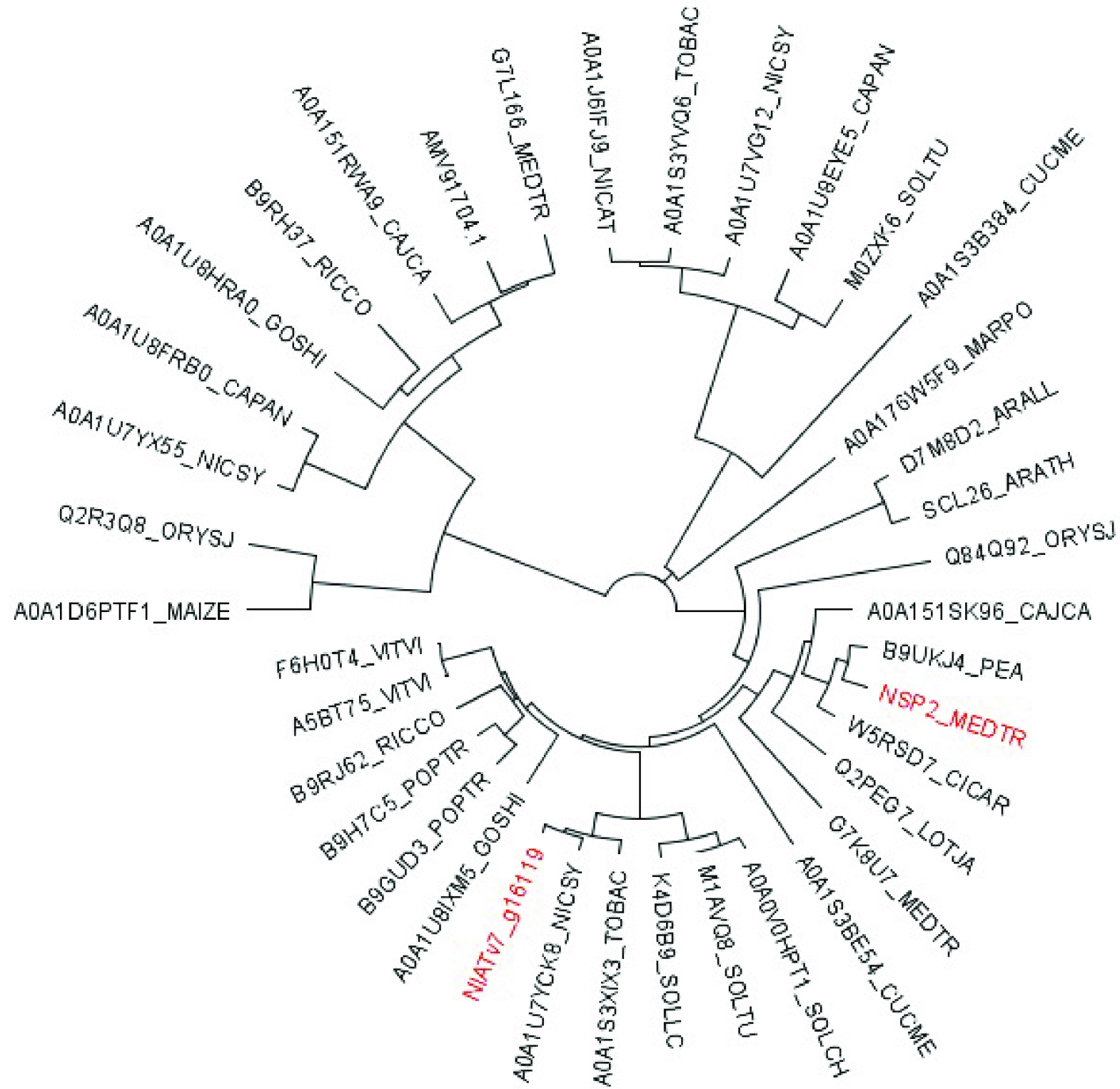

Supplement: Supplementary file 6 — Figure S1. Phylogenetic analysis of NIATv7_g16119, similar to nodulation signaling protein 2 in Medicago (both in red), a putative target of miR171b-3p_1, compared to orthologues in different plant species [69] and to another GRAS transcription factor (AMVG91704.1, RAM1) known from Lotus japonicus to be important for AMF colonization [18]. The tree was constructed with Genious tree builder using the Jukes-Cantor genetic distance model and the neighbor-joining tree builder based on the amino acid sequences. TOBAC – Nicotiana tabacum, NICSY – Nicotiana sylvestris, SOLTU – Solanum tuberosum, NICAT – Nicotiana attenuata, ARATH – Arabidopsis thaliana, POPTR – Populus trichocarpa, ORYSJ- Oryza sativa, GOSHI – Gossypium hirsutum, MEDTR – Medicago truncatula, PEA – Pisum sativum, CAJCA – Cajanus cajan, RICCO – Ricinus communis, CAPAN – Capsicum annuum, SOLCH – Solanum chacoense, SOLLC - Solanum lycopersicum, CUCUME – Cucumis sativus, ARALL – Arabidopsis lyrata, MARPO – Marchantia polymorpha, (PDF 1754 kb) [file 12864_2018_5338_MOESM6_ESM.pdf]
