# Supplementary material for: Complex regulation of microRNAs in roots of competitively-grown isogenic Nicotiana attenuata plants with different capacities to interact with arbuscular mycorrhizal fungi
Source: BMC Genomics. 2018 Dec 17;19:937. doi: 10.1186/s12864-018-5338-x (PMC6296096; doi:10.1186/s12864-018-5338-x)
Supplement: Supplementary file 7 — Table S6. miRNAs differentially expressed in this study with (+) and without (−) AMF inoculum in the two genotypes (empty vector [EV] and irCCaMK) and their expression and function described in literature. (DOCX 109 kb) [file 12864_2018_5338_MOESM7_ESM.docx]

| miRNA family | Function | Reported expression in roots | Putative targets | Species | Refe-rence | EV- | EV+ | irCCaMK- | | irCCaMK+ | |
| --- | --- | --- | --- | --- | --- | --- | --- | --- | --- | --- | --- |
| miR156 | flowering time, fungal infection, P-deficiency, | up-regulated after mycLCO treatment, lateral root formation, regulation of soybean nodulation | SBP-, SPL | *Arabidopsis thaliana, Medicago truncatula, Glycine max* | [1-4] | + | + | - | ++ | |  |
| miR160 | root cap formation, gravity sensing, adventitious rooting, symbiotic nitrogen fixation, root elongation, root hair formation; | down-regulation during AMF formation in tomato; downregulated during nodulation | ARF10, ARF17, ARF16 | *A. thaliana, M. truncatula,*  *Lupinus albus,*  *G. max,*  *Lycopersicum esculentum* | [5-9] | +++ | + | + | + | |  |
| miR162 | mechanical and water stress, | down-regulated after P-starvation | DCL1 | *M. truncatula* | [9, 10] | + | + | ++ | + | |  |
| miR164 | shoot apical meristem formation; lateral root formation; drought stress resistance | upregulation during symbiotic nitrogen fixation | NAC | *Oryza sativa, Lupinus albus, M. truncatula, G. max* | [4, 11, 12] [13] | ++ | (+) | ++ | + | |  |
| miR165-3p  miR165a-5p | meristem size, vasculature differentiation (plant development), reduced lateral root formation and root development; | weak u-pregulation after Myc-factor treatment; | classIII homeodomain leucine zipper transcription factors;  regulatory peptides | *A. thaliana,*  *M. truncatula,* | [2, 14, 15] | ++  - | +  + | ++  - | +  - | |  |
| miR167 | pathogen defense, symbiotic nitrogen fixation, high N, | Up-regulated after AMF treatment, up-regulated after mycLCO treatment | ARF6, ARF8/IAR3 | *A. thaliana*  *L. esculentum,*  *M. truncatula, G. max* | [9, 16-18] | ++ | ++ | +++ | + | |  |
| miR168 | homeostasis and feedback regulation on miRNA, | increase after Myc factor treatment | AGO1 | *A. thaliana, M. truncatula* | [2, 19] | ++ | ++ | ++ | (+) | |  |
| miRNA family | Function | Reported expression in roots | Putative targets | Species | Refe-rence | EV- | EV+ | irCCaMK- | | irCCaMK+ | |
| miR170 | regulation of Al-stress | Up-regulated during nodulation | SCARECROW-like protein transcription factor) | *Arabidopsis,*  *Phaseolus vulgaris* | [20, 21] | ++ | + | + | - | |  |
| miR171 | strong induction under P-sufficient conditions; lateral root formation, root development, enables arbuscule formation | highly expressed in mature nodules, with AMF positive correlation between miR171h and NSP; positive regulation of LOM1, | Transcription factors/NSP2, LOM1 | *M. truncatula; L. esulentum* | [15, 17, 18, 22, 23] | + | +++ | + | +(+) | |  |
| miR172 | ethylene response pathway, N-remobilization; regulated by miR156 | Regulation of nodulation | APETALA2-like transcription factors | *Arabidopsis, G. max, Lotus japonicus, Helianthus annuus* | [1, 24, 25] | + | +++ | + | + | |  |
| miR1878 | expressed in developing rice grains | Not described | -- | *O. sativa* | [26] | + | (+) | + | ++ | |  |
| miR2111 | P-starvation induced (see 399); down-regulated by C, N and S deficiency | Shoot control of nodulation | Kelch-repeat containing protein; symbiosis repressor TML | *A. thaliana, Lotus japonicus* | [27, 28] [29] | + | + | ++ | ++ | |  |
| miR390 | root development; | weak upregulation after Myc factor treatment |  | *M. truncatula; A. thaliana* | [2, 30, 31] | - | + | - | - | |  |
| miR319 | plant growth and morphogenesis; control of JA biosynthesis and senescence, increase during P-deficiency; cold stress | weak up-regulation after Myc treatment and after AMF colonization | TCP transcription factor | *A. thaliana, L. esculentum,* Saccharum sp.  *M. truncatula* | [2, 4, 32-36] | + | ++ | + | +(+) | |  |

| miRNA family | Function | Reported expression in roots | Putative targets | Species | Refe-rence | EV- | EV+ | irCCaMK- | | irCCaMK+ | |
| --- | --- | --- | --- | --- | --- | --- | --- | --- | --- | --- | --- |
| miR393a-5p  miR393a-3p | response to nitrate availability, primary and lateral root growth, antibacterial resistance, response to drought stress; early nodulation  up-regulation of resistance pathways | regulates mycorrhization | TIR1/AFB, F-box proteins and bHLH transcription factors | *A. thaliana*  *M. truncatula,L. esculentum* | [27, 28, 33, 37, 38] | +  +(+) | +  + | +  ++ | ++  + | |  |
| miR398 | pathogen infection, P-starvation related, down-regulated by C, N and S starvation | down-regulated during symbiosis, | copper superoxide dismutases cytochrome C oxidase subunit V | *A. thaliana, G. max, L. esculentum, P. vulgaris* | [6, 27, 28, 38-40] | ++ | + | ++ | + | |  |
| miR399 | up-regulated under Pi deficiency in leaves | locally up-regulated after AMF colonization | Pho2 | *A. thaliana, M. truncatula; L. esculentum* | [28, 33, 41] | ++ | + | ++ | +++ | |  |
| miR403 | antiviral; increased stress resistance | down-regulated after drought stress in roots | AGO2 | *Arabidopsis,*  *H. annuus* | [25, 42] | ++ | +++ | ++ | + | |  |
| miR473 | Upregulated after drought stress; downregulated after wounding |  | UVR8 | *Populus trichocarpa; Aquilaria sinensis* | [43, 44] | + | ++ | + | + | |  |
| miR482 | repressed during pathogen infection, disease-resistance related  / drought stress regulated | Up-regulated during symbiosis | NBS-LRR | *Nicotiana tabacum* | [17, 39, 45, 46] | ++ | + | + | (+) | |  |
| miR6021 | Tobacco-specific; related to R-gene recognition |  | R-genes | *Nicotiana tabacum* | [47] | - | - | ++ | + | |  |

-: not expressed; (+): weak(er) expression compared to +, +: expressed , ++: higher expression compared to the other genotype/treatment

+++: much higher expression

**References**

1. Yan, Z., et al., *miR172 regulates soybean nodulation.* Molecular Plant-Microbe Interactions, 2013. **26**(12): p. 1371-1377.

2. Formey, D., et al., *The small RNA diversity from Medicago truncatula roots under biotic interactions evidences the environmental plasticity of the miRNAome.* Genome Biology, 2014. **15**(9): p. 457.

3. Yu, N., et al., *The role of miR156/SPLs modules in Arabidopsis lateral root development.* The Plant Journal, 2015. **83**(4): p. 673-685.

4. Mao, G., et al., *miR393 and miR164 influence indeterminate but not determinate nodule development.* Plant Signaling & Behavior, 2013. **8**(10): p. e26753.

5. Zhang, W., et al., *Bacteria-responsive microRNAs regulate plant innate immunity by modulating plant hormone networks.* Plant Molecular Biology, 2011. **75**(1-2): p. 93-105.

6. Bustos-Sanmamed, P., et al., *Overexpression of miR160 affects root growth and nitrogen-fixing nodule number in Medicago truncatula.* Functional Plant Biology, 2013. **40**(12): p. 1208-1220.

7. Jin, W. and F. Wu, *Characterization of miRNAs associated with Botrytis cinerea infection of tomato leaves.* BMC Plant Biology, 2015. **15**(1): p. 1.

8. Couzigou, J.M. and J.P. Combier, *Plant microRNAs: key regulators of root architecture and biotic interactions.* New Phytologist, 2016. **212**(1): p. 22-35.

9. Devers, E.A., et al., *Stars and symbiosis: MicroRNA- and microRNA*-mediated transcript cleavage involved in arbuscular mycorrhizal symbiosis.* Plant Physiology, 2011. **156**(4): p. 1990-2010.

10. Capitão, C., et al., *In Medicago truncatula, water deficit modulates the transcript accumulation of components of small RNA pathways.* BMC Plant Biology, 2011. **11**: p. 79-79.

11. Fang, Y., K. Xie, and L. Xiong, *Conserved miR164-targeted NAC genes negatively regulate drought resistance in rice.* Journal of Experimental Botany, 2014. **65**(8): p. 2119-2135.

12. Zhou, M., et al., *Degradome sequencing reveals endogenous small RNA targets in rice (Oryza sativa L. ssp. indica).* Frontiers in Biology, 2010. **5**(1): p. 67-90.

13. Zhu, Y.Y., et al., *microRNA expression profiles associated with phosphorus deficiency in white lupin (Lupinus albus L.).* Plant Science, 2010. **178**(1): p. 23-29.

14. Carlsbecker, A., et al., *Cell signalling by microRNA165/6 directs gene dose-dependent root cell fate.* Nature, 2010. **465**(7296): p. 316-321.

15. Lauressergues, D., et al., *Primary transcripts of microRNAs encode regulatory peptides.* Nature, 2015. **520**(7545): p. 90-U205.

16. Wang, Y., et al., *MicroRNA167-directed regulation of the auxin response factors GmARF8a and GmARF8b is required for soybean nodulation and lateral root development.* Plant Physiology, 2015. **168**(3): p. 984-999.

17. Bazin, J., et al., *Complexity of miRNA-dependent regulation in root symbiosis.* Philosophical Transactions of the Royal Society B-Biological Sciences, 2012. **367**(1595): p. 1570-1579.

18. Wu, P., et al., *Identification of arbuscular mycorrhiza (AM)-responsive microRNAs in tomato.* Frontiers in Plant Science, 2016. **7**(429).

19. Baldrich, P., et al., *Small RNA profiling reveals regulation of Arabidopsis miR168 and heterochromatic siRNA415 in response to fungal elicitors.* BMC Genomics, 2014. **15**(1): p. 1083.

20. Rhoades, M.W., et al., *Prediction of plant microRNA targets.* Cell, 2002. **110**(4): p. 513-520.

21. Mendoza-Soto, A.B., et al., *Responses of symbiotic nitrogen-fixing common bean to aluminum toxicity and delineation of nodule responsive microRNAs.* Frontiers in Plant Science, 2015. **6**(587).

22. Hofferek, V., et al., *MiR171h restricts root symbioses and shows like its target NSP2 a complex transcriptional regulation in Medicago truncatula.* BMC Plant Biology, 2014. **14**(1): p. 199.

23. Couzigou, J.-M., et al., *Positive gene regulation by a natural protective miRNA enables arbuscular mycorrhizal symbiosis.* Cell Host & Microbe, 2017. **21**(1): p. 106-112.

24. Holt, D.B., et al., *micro RNA 172 (miR172) signals epidermal infection and is expressed in cells primed for bacterial invasion in Lotus japonicus roots and nodules.* New Phytologist, 2015. **208**(1): p. 241-256.

25. Ebrahimi Khaksefidi, R., et al., *Differential expression of seven conserved microRNAs in response to abiotic stress and their regulatory network in Helianthus annuus.* Frontiers in Plant Science, 2015. **6**: p. 741.

26. Nguyen, G.N., et al., *Role of microRNAs involved in plant response to nitrogen and phosphorous limiting conditions.* Frontiers in Plant Science, 2015. **6**: p. 629.

27. Hsieh, L.C., et al., *Uncovering small RNA-mediated responses to phosphate deficiency in Arabidopsis by deep sequencing.* Plant Physiology, 2009. **151**(4): p. 2120-2132.

28. Liang, G., Q. Ai, and D. Yu, *Uncovering miRNAs involved in crosstalk between nutrient deficiencies in Arabidopsis.* Scientific Reports, 2015. **5**: p. 11813.

29. Tsikou, D., et al., *Systemic control of legume susceptibility to rhizobial infection by a mobile microRNA.* Science, 2018.

30. Yoon, E.K., et al., *A molecular framework for the differential responses of primary and lateral roots to auxin in Arabidopsis thaliana.* Journal of Plant Biology, 2014. **57**(5): p. 274-281.

31. Cabrera, J., et al., *Differentially expressed small RNAs in Arabidopsis galls formed by Meloidogyne javanica: a functional role for miR390 and its TAS3-derived tasiRNAs.* New Phytologist, 2016. **209**(4): p. 1625-1640.

32. Schommer, C., et al., *Control of jasmonate biosynthesis and senescence by miR319 targets.* Plos Biology, 2008. **6**(9): p. 1991-2001.

33. Gu, M., et al., *Expression analysis suggests potential roles of microRNAs for phosphate and arbuscular mycorrhizal signaling in Solanum lycopersicum.* Physiologia Plantarum, 2010. **138**(2): p. 226-237.

34. Zhao, W., et al., *Identification of jasmonic acid-associated microRNAs and characterization of the regulatory roles of the miR319/TCP4 module under root-knot nematode stress in tomato.* Journal of Experimental Botany, 2015. **66**(15): p. 4653-4667.

35. Zhang, F., et al., *Phosphorylation of CBP20 links microRNA to root growth in the ethylene response.* PLoS Genetics, 2016. **12**(11): p. e1006437.

36. Thiebaut, F., et al., *Regulation of miR319 during cold stress in sugarcane.* Plant, Cell & Environment, 2012. **35**(3): p. 502-512.

37. Vidal, E.A., et al., *Nitrate-responsive miR393/AFB3 regulatory module controls root system architecture in Arabidopsis thaliana.* Proceedings of the National Academy of Sciences, 2010. **107**(9): p. 4477-4482.

38. Etemadi, M., et al., *Auxin perception is required for arbuscule development in arbuscular mycorrhizal symbiosis.* Plant Physiology, 2014. **166**: p. 281-292.

39. Naya, L., et al., *Regulation of copper homeostasis and biotic interactions by microRNA 398b in common bean.* PLOS ONE, 2014. **9**(1): p. e84416.

40. Khraiwesh, B., J.K. Zhu, and J.H. Zhu, *Role of miRNAs and siRNAs in biotic and abiotic stress responses of plants.* Biochimica Et Biophysica Acta-Gene Regulatory Mechanisms, 2012. **1819**(2): p. 137-148.

41. Branscheid, A., et al., *Expression pattern suggests a role of miR399 in the regulation of the cellular response to local Pi increase during arbuscular mycorrhizal symbiosis.* Molecular Plant-Microbe Interactions, 2010. **23**(7): p. 915-926.

42. Harvey, J.J.W., et al., *An antiviral defense role of AGO2 in plants.* PLoS ONE, 2011. **6**(1): p. e14639.

43. Shuai, P., et al., *Identification of drought-responsive and novel Populus trichocarpa microRNAs by high-throughput sequencing and their targets using degradome analysis.* BMC Genomics, 2013. **14**: p. 233-233.

44. Gao, Z.-H., et al., *Profiling of microRNAs under wound treatment in Aquilaria sinensis to identify possible microRNAs involved in agarwood formation.* International Journal of Biological Sciences, 2014. **10**(5): p. 500-510.

45. Li, H., et al., *Misexpression of miR482, miR1512, and miR1515 increases soybean nodulation.* Plant Physiology, 2010. **153**(4): p. 1759-1770.

46. Shivaprasad, P.V., et al., *A microRNA superfamily regulates nucleotide binding site–leucine-rich repeats and other mRNAs.* The Plant Cell, 2012. **24**(3): p. 859-874.

47. Li, F., et al., *MicroRNA regulation of plant innate immune receptors.* Proceedings of the National Academy of Sciences of the United States of America, 2012. **109**(5): p. 1790-1795.
